# Case Report: sustained five-year remission in eosinophilic granulomatosis with polyangiitis with intestinal perforation after surgery and rituximab-based therapy without glucocorticoid escalation

**DOI:** 10.3389/fimmu.2026.1849453

**Published:** 2026-07-01

**Authors:** Yuriko Yamamura, Yoshinori Matsumoto, Keiji Ohashi, Keigo Hayashi, Yoshia Miyawaki, Haruki Watanabe, Eri Katsuyama, Takayuki Katsuyama, Mariko Takano-Narazaki, Jun Wada

**Affiliations:** 1 Department of Nephrology, Rheumatology, Endocrinology and Metabolism, Okayama University Faculty of Medicine, Dentistry and Pharmaceutical Sciences, Okayama, Japan; 2 School of Infection and Immunity, University of Glasgow, Glasgow, United Kingdom; 3 Department of Rheumatology, Kurashiki Medical Center, Kurashiki, Japan; 4 Department of Medical Laboratory Science, Okayama University Faculty of Health Sciences, Okayama, Japan

**Keywords:** EGPA, eosinophilic granulomatosis with polyangiitis, gastrointestinal involvement, glucocorticoid sparing, glucocorticoids, perioperative management, rituximab, surgery

## Abstract

Eosinophilic granulomatosis with polyangiitis (EGPA) is a systemic necrotizing vasculitis characterized by eosinophilic infiltration and granuloma formation, affecting multiple organs. Gastrointestinal (GI) involvement is relatively uncommon and it typically presents with nonspecific symptoms, such as abdominal pain or diarrhea; in contrast, ulceration and intestinal perforation are rare, but potentially life−threatening complications that often require surgical intervention. The standard treatment for severe GI EGPA includes high−dose glucocorticoids combined with cyclophosphamide or rituximab (RTX). However, the perioperative escalation of glucocorticoids is generally avoided owing to the increased risk of postoperative complications. We report a case with a 5-year follow−up of EGPA resistant to multiple immunosuppressive agents with severe GI involvement, including intestinal perforation and multiple jejunal ulcers, which was successfully treated with RTX for postoperative remission induction and long−term maintenance therapy without any prednisolone escalation. The prednisolone dose was gradually reduced from 17.5 mg/day to 1 mg/day over two years without any escalation, and a sustained remission was achieved throughout the course. This case suggests that RTX may represent a viable therapeutic option for severe or treatment−resistant EGPA in cases with GI involvement when glucocorticoid escalation is undesirable or unsafe, such as in the perioperative setting.

## Introduction

Eosinophilic granulomatosis with polyangiitis (EGPA), a systemic necrotizing vasculitis affecting small- to medium-sized vessels, which is characterized by eosinophil-rich granulomatous inflammation and it is commonly associated with asthma and peripheral eosinophilia (1). Gastrointestinal (GI) involvement occurs in approximately 23% of patients, most often presenting with abdominal pain (20%) (2), whereas severe complications, such as ulcerations or intestinal perforation, are relatively uncommon but clinically significant, as they can be fatal and frequently require surgical intervention (2).

Current treatment recommendations for severe EGPA rely on intensive immunosuppressive therapy combining high-dose glucocorticoids (GC) with cyclophosphamide (CYC) or rituximab (RTX) (3, 4). However, for patients requiring surgery, the escalation of GC doses poses particular challenges, as higher perioperative GC exposure is associated with an increased risk of postoperative complications, including infections and anastomotic leakage (5). This creates a therapeutic dilemma in patients with severe EGPA and GI involvement who require surgical management. Generally, high-dose GC is considered to be the standard induction therapy but may be unsafe in the perioperative setting. To date, no studies have specifically addressed this issue.

We herein report a severe case of EGPA resistant to multiple immunosuppressive agents, including CYC, who developed intestinal perforation and multiple jejunal ulcers requiring sigmoid colon resection, as confirmed by computed tomography and endoscopy. Because GC escalation should be avoided in the perioperative period, RTX was initiated without increasing the GC dose. Despite the relatively low GC exposure, RTX successfully induced remission and enabled sustained long-term disease control for over five years at a minimal GC dose. This case highlights the potential usefulness of RTX as an effective GC-sparing induction and maintenance therapy capable of supporting durable long-term remission without GC escalation.

## Case description

A 63-year-old man presented to a previous hospital with a two-month history of urticaria and purpura, a one-month history of persistent nocturnal cough, and peripheral eosinophilia. He subsequently developed progressive sensory impairment and motor dysfunction in both feet, which was diagnosed as mononeuropathy multiplex, and was admitted for further evaluation to the previous general hospital in December 2016. Laboratory tests showed marked eosinophilia (24702/µL) and elevated C-reactive protein (CRP) (12.6 mg/dL), while myeloperoxidase-antineutrophil cytoplasmic antibody (MPO-ANCA) and proteinase 3-ANCA (PR3-ANCA) were negative. Based on the presence of two extrapulmonary manifestations, a history suggestive of asthma, and marked eosinophilia, and the exclusion of alternative diagnoses, EGPA was clinically diagnosed by a rheumatologist according to the Lanham criteria (6). Pulmonary, cardiac, and gastrointestinal involvements were not identified on further investigation, included radiographic and endoscopic investigations. Prednisolone (60 mg/day, approximately 1 mg/kg/day) was initiated as the remission induction therapy. Although his neurologic symptoms improved, both eosinophilia and inflammatory markers recurred during GC tapering.

**Figure 1:**
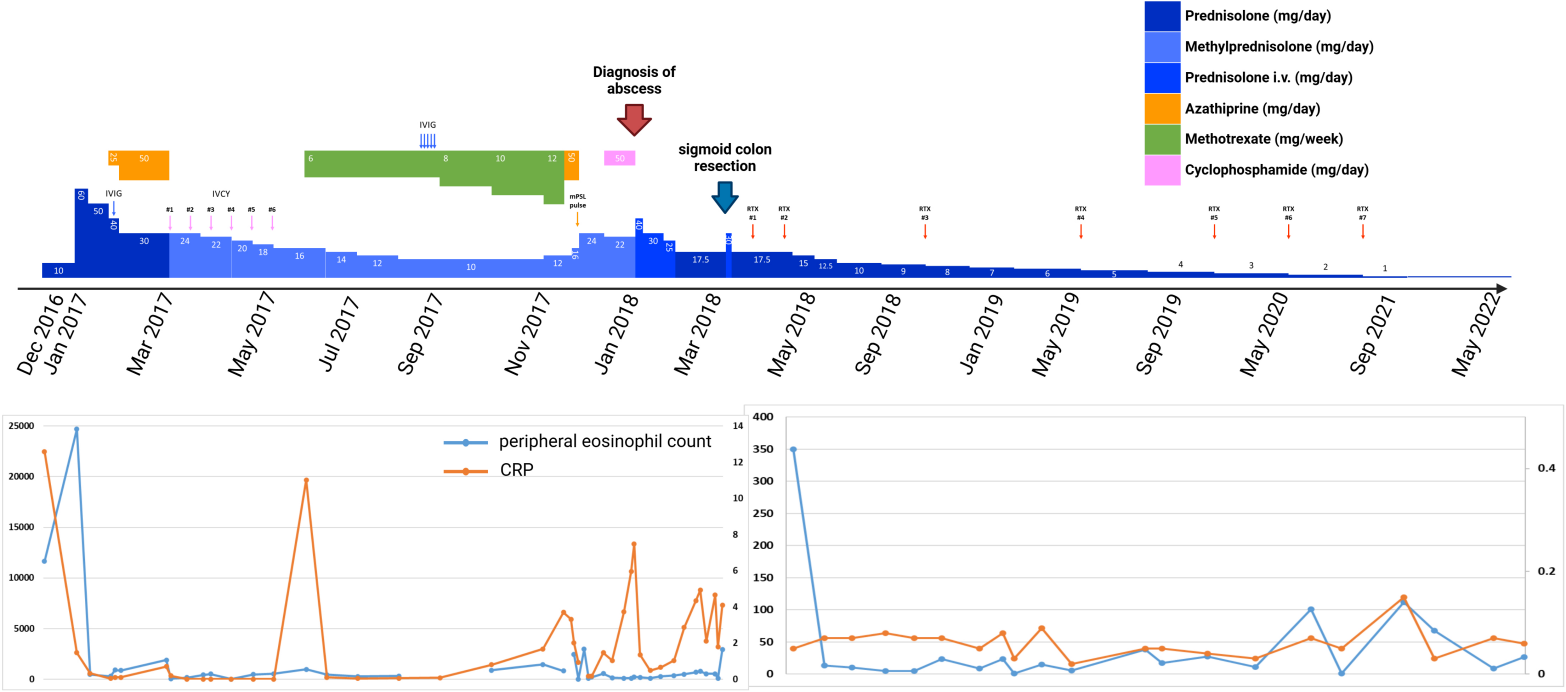
Clinical course and treatment timeline over the entire observation period. The timeline shows glucocorticoid (GC) doses, conventional immunosuppressive agents, sigmoid colon resection, rituximab (RTX) induction and maintenance infusions, peripheral eosinophil counts, and C-reactive protein (CRP) levels at key assessable time points. The treatment course illustrates the difficulty in achieving sustained remission and tapering GCs despite the combination of multiple immunosuppressive therapies prior to RTX induction. Disease activity, assessed by peripheral eosinophil count and inflammatory marker, CRP levels, fluctuated during this period. Following sigmoid colon resection and subsequent RTX induction, both eosinophil counts and CRP levels were rapidly suppressed at subsequent clinical assessments, allowing GCs tapering without escalation or addition of other conventional immunosuppressants.

To facilitate further GC tapering, multiple immunosuppressive agents, including azathioprine (AZA), intravenous immunoglobulin (IVIG), methylprednisolone pulse therapy (mPSL, 500 mg/day for 3 days), intravenous CYC (500 mg monthly for 6 cycles), and methotrexate (MTX, up to 12 mg/week), were sequentially introduced. Despite these treatments, both the eosinophilia and elevated CRP levels reappeared with each attempt to taper GC. Therefore, the patient was referred to our hospital for further management in December 2017. On admission, the patient was receiving only methylprednisolone (22 mg/day) and was asymptomatic, except for right-hand numbness, which was later diagnosed to be carpal tunnel syndrome. Nonetheless, the eosinophil and CRP elevation persisted, and oral CYC (50 mg/day) was initiated (**Figure 1**).

**Figure 2:**
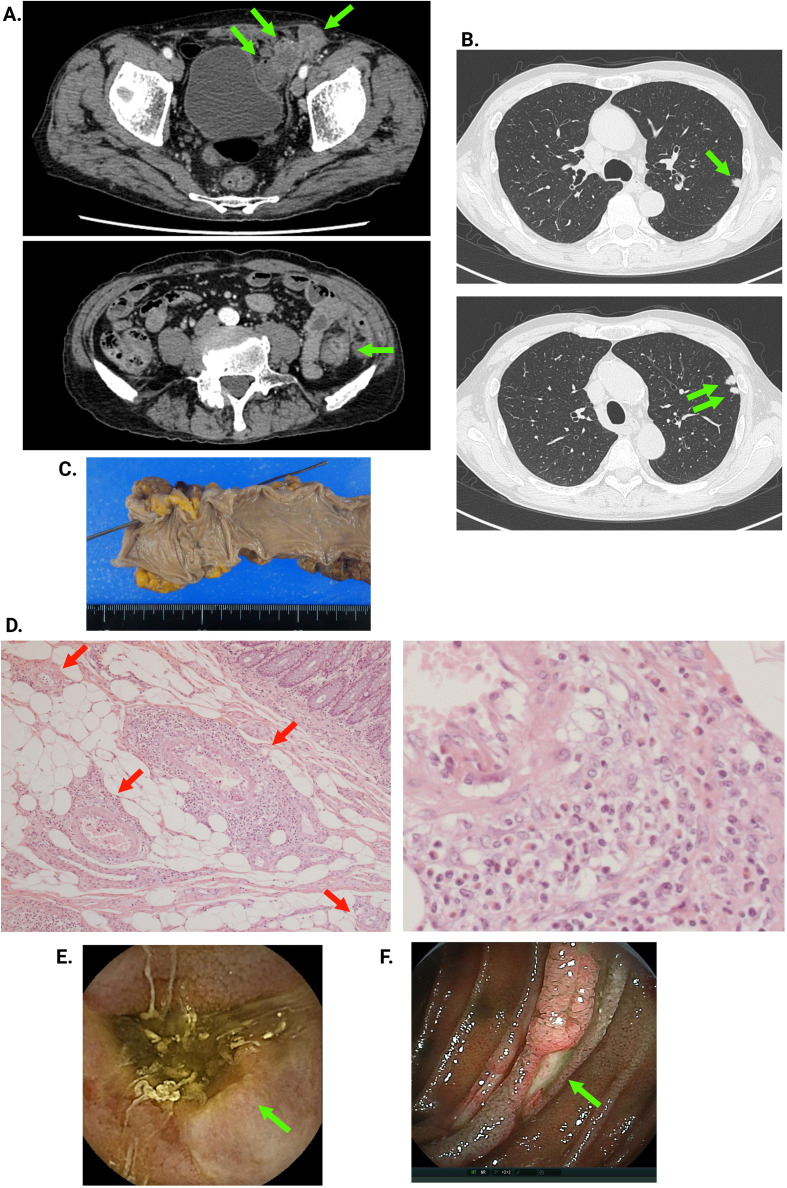
Imaging, histopathology, and endoscopic findings. **(A)** Contrast-enhanced abdominal CT image showing a left inguinal and descending colon abscess adjacent to sigmoid colon (arrows). **(B)** Non-contrast-enhanced chest CT images showing multiple nodular and subpleural consolidative lesions in the left lung (arrows). **(C)** Resected specimen of the resected sigmoid colon showing the perforation site. **(D)** Hematoxylin and Eosin staining of the resected sigmoid colon showing vasculitis with fibrinoid necrosis extending from the submucous to subserous layers, with eosinophil-rich and lymphocytic inflammatory infiltration (arrows). **(E)** Capsule endoscopy before rituximab (RTX) induction demonstrating multiple jejunum ulcers (arrows). **(F)** Double-balloon endoscopy performed twenty days after RTX induction showing marked improvement of the jejunal ulcers (arrows). Arrows indicate the key findings in each panel.

In January 2018, the patient developed a painful mass in the left lower abdomen. Computed tomography (CT) revealed a left inguinal and retroperitoneal abscess adjacent to the sigmoid colon, raising suspicion of an occult intestinal perforation (**Figure 2A**), along with multiple irregular subpleural consolidations accompanied by scattered nodular and ground-glass lesions opacities (**Figure 2B**). Definite extraluminal free air or contrast extravasation was not clearly identified on the representative CT images. The abscess was initially managed with percutaneous drainage and antibiotics, along with a gradual reduction in the GC dosage to prednisolone 17.5 mg/day. However, the abscess enlarged when the drain was clamped, prompting surgical intervention. In March, the patient underwent sigmoid colon resection for infection control and diagnostic clarification. Intestinal perforation site was identified in the resected specimen (**Figure 2C**). Histopathological examination showed vasculitis with fibrinoid necrosis extending from the submucosa to the subserosa, surrounded by prominent eosinophil-rich and lymphocyte infiltration, without granuloma formation. Although granulomas were absent, the presence of eosinophilic vasculitis and perivascular eosinophil-dominant infiltration is consistent with highly active EGPA with gastrointestinal involvement, meeting both the Lanham criteria and the American College of Rheumatology 1990 Criteria (6, 7) (**Figure 2D**). Capsule endoscopy performed after surgery revealed multiple jejunal ulcers formation, indicating high disease activity with a Birmingham Vasculitis Activity Score (BVAS) of 9 (8), necessitating intensive remission induction therapy in addition to the surgical management (**Figure 2E**).

Considering that the disease was resistant to conventional immunosuppressive therapy and that GCs tapering below 10 mg/day was not achievable, along with the substantial risk of postoperative infectious and protracted wound healing complications associated with GC escalation, RTX was selected as a remission induction strategy. Two doses of RTX (1000 mg) were administered three weeks apart, starting 20 days after surgery, without increasing the prednisolone dose from 17.5 mg/day. Mepolizumab and benralizumab were not considered therapeutic options because they had not yet been approved for the treatment of EGPA in Japan at that time.

Twenty days after the first RTX infusion, double-balloon endoscopy confirmed a significant improvement in the jejunal ulcers (**Figure 2D**). A CT scan did not show exacerbation of the pulmonary manifestations, thus the patient was therefore considered to have achieved BVAS 0 at this point. The patient subsequently made a full recovery from surgery and has since completed a 5-year follow-up in our outpatient clinic. During this period, BVAS remained at 0, defined as remission, and prednisolone was gradually tapered to 1 mg/day over two years without any GC dose escalation (**Figure 1**). Maintenance therapy with RTX (1000 mg every six months) was continued, and a sustained remission has been maintained for five years without either recurrence or any treatment-related adverse effects.

## Discussion

EGPA is an antineutrophil cytoplasmic antibody (ANCA)-associated vasculitis characterized by eosinophil infiltration and multi-organ involvement, most commonly affecting the lungs (91%), peripheral nerves (55%), and the ear, nose, and throat (ENT) regions (48%). GI involvement is less frequent, occurring in approximately 23% of all patients, and only a small subset (6%) develops severe complications requiring intervention, such as intestinal ulcers or perforation (2). Although uncommon, severe GI manifestations are associated with markedly increased mortality, reported to be as high as 55% (2, 9) and are therefore incorporated into the Five-Factor Score (FFS) (10, 11). These data underscore the need for early and aggressive induction therapy in EGPA patients with GI involvement.

RTX was introduced for the treatment of ANCA-associated vasculitis in the 2010s (12, 13), and its efficacy in EGPA has been supported by retrospective analyses (14). Based on these studies, the American College of Rheumatology (ACR)/Vasculitis Foundation Guideline recommends high-dose GC combined with CYC or RTX over mepolizumab, humanized anti-interleukin-5 (IL-5) monoclonal antibody, for remission induction in active and severe EGPA (4). Thus, high-dose GC, including intravenous pulse therapy, remains strongly recommended for cases with life-threatening organ involvement (4).

More recently, the REOVAS randomized trial demonstrated that RTX is not inferior to standard therapy with GC plus CYC for remission induction in severe EGPA (FFS≧1) over a 12-month period (15). While REOVAS supports the role of RTX in induction therapy, all participants received high-dose GC (up to 1 mg/kg/day), while leaving unanswered the question of whether RTX itself can induce remission under a GC-sparing strategy. Additionally, because the follow-up period was limited to 12 months, the long-term durability of RTX-induced remission and its GC-sparing potential over extended periods remain uncertain.

Our patient presented with severe GI involvement, but lacked ENT manifestations, thus resulting in a Five-Factor Score (FFS) of 2, corresponding to a previously reported two-year survival rate below 0.6 (11). This prognostic estimation supported the need for intensive immunosuppressive therapy. Considering the failure of previous treatments, including AZA, IVIG, MTX, and both intravenous and oral CYC, we selected RTX to improve the likelihood of disease control. The alternative approach considered was escalation of GC dosage from the ongoing 17.5 mg/day, however, this was considered high risk in the perioperative context, where high-dose GCs are associated with increased risks of postoperative infection and wound-healing complications (5). Evidence to guide RTX induction in the immediate postoperative setting in EGPA is extremely limited. Although RTX is not routinely introduced in the early postoperative period, a limited parallel may be drawn from ABO-incompatible kidney transplantation, one of the few clinical contexts in which RTX has been administered in close temporal proximity to major surgery (16). In this setting, RTX has been incorporated into perioperative immunosuppressive protocols without a clear signal for increased postoperative complications, while achieving effective immune control (17, 18). In a randomized, double-blind study, RTX demonstrated a favorable profile, providing effective immunosuppression with a trend toward low rates of acute rejection without an increase in postoperative complications, such as infections, compared to non-RTX regimens (18). However, given the substantial differences in patient populations, surgical context, and background immunosuppression, this analogy should be interpreted with caution and cannot be directly extrapolated to EGPA. In the present case, the use of RTX was therefore guided primarily by clinical necessity, including refractory disease activity and the need to avoid GCs escalation, rather than by established evidence in comparable postoperative settings.

The subsequent clinical course supports this feasibility of this approach. The patient experienced no perioperative complications, including surgical or infectious aspects, while achieving not only marked improvement in disease activity, but also reduction in GC dose that had not previously been achievable. Remarkably, significant improvement was observed after RTX initiation, and the patient subsequently maintained remission. Over the following five years, disease control was sustained with RTX maintenance therapy and progressive GC tapering to 1 mg/day, without any recurrence or treatment-related complications.

This case presented a combination of rare and challenging features, including severe gastrointestinal involvement, treatment-resistant EGPA with failure of multiple immunosuppressive agents (including CYC), and a high-risk perioperative setting in which escalation of GC was best avoided. The successful induction and long-term maintenance of remission with RTX, despite the prior failure of several therapies, suggests that RTX may serve as a potent therapeutic option for EGPA with gastrointestinal involvement, even when high-dose GC cannot be safely administered. However, based on a single case, further accumulation of similar cases is warranted to strengthen this evidence. This case report has several limitations. First, as a single observational case, no causal relationship can be established between RTX and the favorable clinical outcome. The improvement in gastrointestinal lesions and long-term disease control may have been influenced by multiple factors, including surgical resection of the perforated intestinal lesion, percutaneous drainage, antibiotic therapy, postoperative management, bowel rest and nutritional support, and ongoing prednisolone and prior immunosuppressive treatment. Therefore, the individual contribution of RTX cannot be disentangled. Second, RTX was not administered as true monotherapy, but in combination with moderate dose of prednisolone at RTX initiation and continued low-dose glucocorticoids during maintenance therapy. Third, the rapid endoscopic improvement observed 20 days after the first RTX infusion should be interpreted with cautious, as this early time point does not establish a direct effect of RTX. Further accumulation of similar cases is required to clarify the efficacy, safety, and optimal timing of RTX-based therapy in severe gastrointestinal EGPA requiring surgical intervention.

In conclusion, this case illustrates a favorable five-year clinical course of severe gastrointestinal EGPA after surgical source control followed by RTX-based induction and maintenance therapy without glucocorticoid escalation. Although the role of RTX cannot be isolated from the effects of surgery, infection control, and postoperative management, RTX-based treatment may be considered as a potential glucocorticoid-sparing option in selected patients with severe or treatment-resistant EGPA when glucocorticoid escalation is undesirable. Further case accumulation is needed to determine the efficacy and safety of this approach.

## Patient perspective

The patient explained that his greatest source of anxiety was not knowing which organ was affected or why the disease remained active despite the absence of any clear symptoms. Learning that he had an intestinal perforation was shocking. However, finally identifying the focus of the vasculitis also brought relief by providing a clear explanation for his condition. After remission induction with rituximab, his anxiety significantly decreased, and he felt that he was returning to his usual state of health.

## Data Availability

The original contributions presented in the study are included in the article/supplementary material. Further inquiries can be directed to the corresponding author.
